# Declining soil Crustacea in a World Heritage Site caused by land nemertean

**DOI:** 10.1038/s41598-017-12653-4

**Published:** 2017-09-29

**Authors:** Shotaro Shinobe, Shota Uchida, Hideaki Mori, Isamu Okochi, Satoshi Chiba

**Affiliations:** 1 Graduate School of Life Science & Center for Northeast Asian Studies, Tohoku University, 41 Kawauchi, Aoba-ku, Sendai, Miyagi 980–8576 Japan; 2 Japan Wildlife Research Center, 3-3-7, Kotobashi, Sumida, Tokyo 130–8606 Japan; 3 Japan Forest Technology Association, 7, Rokubancho, Chiyoda-ku, Tokyo 102-0085 Japan

## Abstract

Invasive non-native species are of great concern throughout the world. Potential severity of the impacts of non-native species is assessed for effective conservation managements. However, such risk assessment is often difficult, and underestimating possible harm can cause substantial issues. Here, we document catastrophic decline of a soil ecosystem in the Ogasawara Islands, a UNESCO World Heritage site, due to predation by non-native land nemertine *Geonemertes pelaensis* of which harm has been previously unnoticed. This nemertine is widely distributed in tropical regions, and no study has shown that it feeds on arthropods. However, we experimentally confirmed that *G. pelaensis* predates various arthropod groups. Soil fauna of Ogasawara was originally dominated by isopods and amphipods, but our surveys in the southern parts of Hahajima Island showed that these became extremely scarce in the areas invaded by *G. pelaensis*. Carnivorous arthropods decreased by indirect effects of its predation. Radical decline of soil arthropods since the 1980s on Chichijima Island was also caused by *G. pelaensis* and was first recorded in 1981. Thus, the soil ecosystem was already seriously damaged in Ogasawara by the nemertine. The present findings raise an issue and limitation in recognizing threats of non-native species.

## Introduction

Invasive non-native species transported by human activities have caused serious impacts on native ecosystems^1–3^. Accurate risk assessment of the non-native species is essential for successful ecosystem management^2^, because not all non-native species cause harm to ecosystems, and only a fraction established have an effect that is considered harmful^3,4^. Black, grey and white lists are used for simplifying the processes to address this issue^5^. A black list is useful to anticipate threats of particular non-native species and to prevent their extension. In contrast, species that are not on the black list are generally perceived as not being threats or at least less threatening. Therefore, underestimation of potential impact may be seriously detrimental in ecosystems by delaying conservation responses or allowing spreads of the harmful invasive species. We provide here a case study providing an insight on this issue.

**Figure 1:**
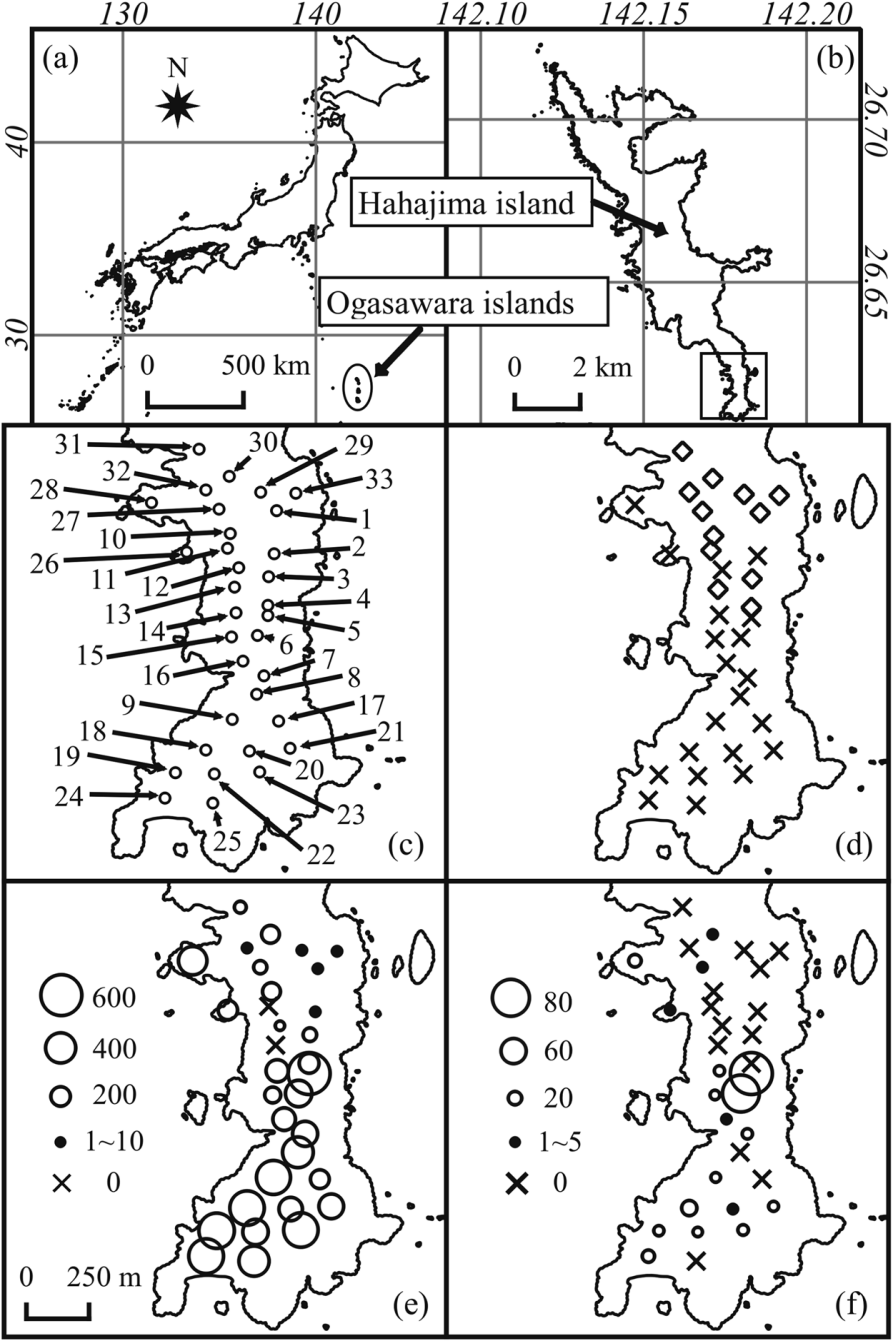
Maps of study site and distributions of *G. pelaensis* and arthropod taxa. (**a**) Location of Ogasawara Islands. (**b**) Location of Hahajima-Island. (**c**) Maps of Minamizaki and study sites. (**d**) Distribution areas of *G. pelaensis* indicated by squares. (**e**,**f**) Occurrence and density of Isopoda (**e**), Amphipoda (**f**). Density is shown in size of circles. Figure 1 was created using the QGIS ver 2.16. Copyright © (1974–2014) National Land Information Division, National Spatial Planning and Regional Policy Bureau, MLIT of Japan. Figure was created and modified by authors based on coastline data of National Land Numerical Information with permission of National Land Information Division, National Spatial Planning and Regional Policy Bureau, MLIT of Japan.

Non-native species are particularly of concern on islands, because invasive non-native species often cause serious decline of island ecosystems^6–9^. Like other oceanic islands, a number of non-native species have been introduced to the Ogasawara archipelagos in the western Pacific ocean (Fig. 1). The Ogasawara archipelagos are registered as a UNESCO World Heritage Site (WHS) because of their unique ecosystems^10^. Conservation programs have been carried out to control the invasive species that have impacted the ecosystem of Ogasawara^11^, such as *Pheidole megacephala* ^12^, *Bufo marinus* ^13^, *Anolis carolinensis* ^14^, *Achatina fulica* ^15^, *Rattus rattus* ^16^ and *Platydemus manokwari* ^17^. In Japan, potential invasiveness was screened for all of the recorded non-native species. The Invasive Alien Species Act, adopted in 2004, specifies that species that are designated as “invasive alien species” (IASs) by the Japan Ministry of the Environment are subject to strict monitoring and that trade in these species is prohibited^18^. Therefore, invaded IASs in Ogasawara have been monitored, eradicated and/or their further spread has been prevented within the islands. For example, *Bufo marinus*, which was introduced to Ogasawara in 1949, has been controlled, because this toad is listed as an IAS and has been believed to have caused serious decline of soil arthropods in Ogasawara^19,20^. Soil animals of Ogasawara were originally composed by arthropods and land snails and particularly dominated by isopods and amphipods^21,22^. On the Chichijima Island, these land crustaceans have radically decreased since the 1980s, and currently they are mostly absent on the island^23,24^. Subsequently, a similar decrease of soil arthropods has occurred on Hahajima Island, and currently isopods and amphipods are abundant only in the southernmost part of the island^25^. However, recent eradication of *B. marinus* on the southern parts of Hahajima resulted in no increases of isopods, but instead, the range of the isopods has continued to contract in the north-to-south direction^25–27^. Thus, *B. marinus* appears not to be a main factor causing the decline of the soil arthropods in Ogasawara^27^.

Recently, invasive African big-headed ants *Pheidole megacephala* were found on Hahajima, and their impacts on land snails were detected^12^. However, the ranges of *P. megacephala* are limited, and these ants are unlikely to be a major cause of the decline of soil arthropods, though surveys are needed to estimate their impacts.

Because of the limitation of resources that can be provided for conservation in Ogasawara, all efforts to control non-native species are focused on the species listed as IASs or those with records of exhibiting invasiveness in other regions, and few surveys are provided for other non-native species. The land nemertine distributed on the Ogasawara Islands is such an example. The land nemertine occurred from the Ogasawara Islands was described as *Geonemertes pelaensis* by Kawakatsu^28^, Okochi^29^ and Kajihara^30^. Several researchers have studied taxonomy and distribution of land nemertine in Ogasawara since 1990s^28–32^. This species is widely distributed in tropical regions as a non-native species, particularly on islands^33–35^. There is no record of impacts on ecosystems caused by *G. pelaensis*. In Ogasawara, this species was first recorded on Chichijima in 1981^28^ and on Hahajima in 1995, and the species is currently distributed in entire parts of Chichijima^31^. A previous study experimentally showed that *G. pelaensis* is malacophagous and does not feed on arthropods^36^. However, in Ogasawara, *G. pelaensis* was not an effective predator on land snails^29^. Hence, this non-native nemertine has been recognized as a species that causes no harm to the ecosystem. However, we speculate that, in fact, this nemertine might be a predator that has caused the catastrophic decline of soil arthropod fauna in Ogasawara. In the present study, we test this hypothesis using feeding experiments and field surveys.

## Results

### Soil arthropod communities

The Ogasawara archipelagos are composed of the Chichijima, Hahajima (Fig. 1b) and Mukojima Islands. The study area was in Minamizaki, which was in the southern part of Hahajima (Fig. 1b,c). Surveys in previous studies^29^ and our preliminary surveys documented that the land nemertine *Geonemertes pelaensis* is distributed throughout mostly all parts of the north and central Hahajima. In the present study, we found that land nemertine is not distributed in the southern part of Minamizaki. In the northern part of Minamizaki, *G. pelaensis* occurred throughout except for a few sites in the central and western parts. The distributional area of *G. pelaensis* did not overlap with the distributional area of the African big-headed ant *P. megacephala*. Among the sites surveyed, *G. pelaensis* existed at 12 sites, and *P. megacephala* were found at 7 sites. Both of *G. pelaensis* and *P. megacephala* were not found at 14 sites.

**Figure 2:**
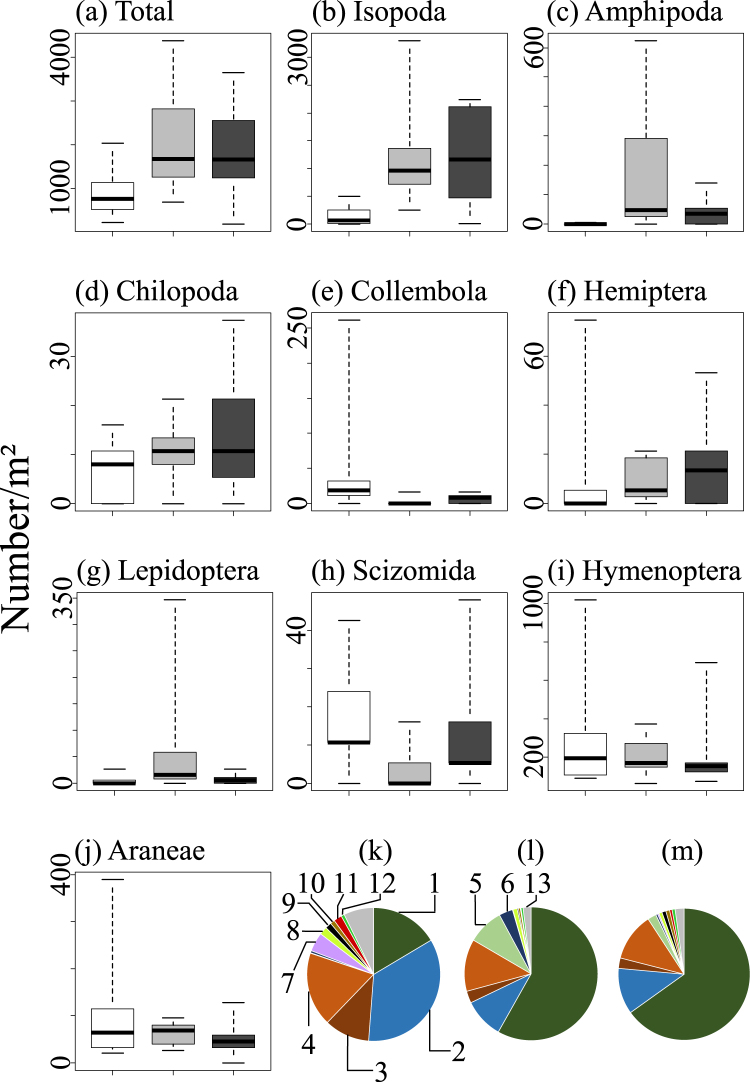
(**a**–**j**) Box plots showing changes in density of each of nine arthropod taxa at the sites where *G. pelaensis* exsit (white), those where *P. megacephala* exist (grey), and those where both *G. pelaensis* and *P. megacephala* are absent (black). Whiskers indicate the minimum and maximum density at each site. Horizontal lines of the box except for thick line indicate 25% and 75% quantile. A thick line indicates mean value. (**k**–**m**) Composition of the arthropod fauna at the sites where *G. pelaensis* exsit (**k**), those where *P. megacephala* exist (**l**), and those where both *G. pelaensis* and *P. megacephala* are absent (**m**). Numbers in pie charts correspond to each taxonomies; Isopoda, 1, Hymenoptera, 2, Araneae, 3, Diplopoda, 4, Amphipoda, 5, Lepidoptera, 6, Collembola, 7, Coleoptera, 8, Diplura, 9, Hemiptera, 10, Scizomida, 11, Chilopoda, 12, Others, 13.

The most dominant order among the soil arthropods in the Minamizaki area was Isopoda (5174 individuals in total, occupying 53.5% of the whole individuals). The average density of Isopoda at the sites where both *G. pelaensis* and *P. megacephala* were absent (1232.8 individuals/m^2^) was mostly equal compared to the average density at the sites where *P. megacephala* were found (1237.3 individuals/m^2^). In contrast, the average density of Isopoda at the sites where *G. pelaensis* were found was only approximately one-tenth of that at the sites where no *G. pelaensis* were found (139.6 individuals/m^2^) (Fig. 2). The ranges of Isopoda with high density were restricted to the areas where *G. pelaensis* was absent (Fig. 1). Especially, the endemic species of *Spherillo*, which dominated in the isopod community of the southern parts of Minamizaki, was absent in most of the sites where *G. pelaensis* was found (Fig. 1). The selected GLMM showed that existence of *G. pelaensis* negatively affected the density of Isopoda (P = 7.68 × 10^−6^) (Supplementary Table S1).

Amphipoda was mostly absent at the sites where *G. pelaensis* was found (Fig. 1). The average density of Amphipoda at the sites where *G. pelaensis* was absent was 40.4 individuals/m^2^ (*P. megacephala* absent) and 185.9 individuals/m^2^ (*P. megacephala* present), whereas it was 0.9 individuals/m^2^ at the sites where *G. pelaensis* was found (Fig. 2). The selected GLMM showed that existence of *G. pelaensis* negatively affected the density of Amphipoda (P = 3.35 × 10^−6^) (Supplementary Table S1).

Effects of *G. pelaensis* on the density of other orders detected in GLMM analyses are shown in Supplementary Table S1. In Chilopoda, density was also negatively affected by the presence of *G. pelaensis* in selected GLMM (P = 3.01 × 10^−2^). In Hemiptera, presence of *G. pelaensis* was not included in selected GLMM, but *Nerthra macrothorax*, which occupied more than 80% of individuals of Hemiptera collected, was affected by presence of *G. pelaensis* (P = 7.10 × 10^−3^) (Supplementary Table S1). Indeed, *N. macrothorax* was mostly absent at the sites where *G. pelaensis* was found (Fig. 1). In other orders such as Diplopoda, Araneae and Hymenoptera, no negative effects of *G. pelaensis* was detected in the GLMM analyses.

Negative impacts of *P. megacephala* on the density of soil arthropods were less obvious than those of *G. pelaensis* (Fig. 2). On the basis of the GLMM analyses, *P. megacephala* provided negative effects on Schizomida (P = 1.24 × 10^−2^) (Supplementary Table S1).

Total biomass represented by total numbers of individuals at the sites where *G. pelaensis* existed was approximately 40% of those at the sites where no *G. pelaensis* was found. Radical differences in the composition of soil arthropod fauna were found between the sites where *G. pelaensis* existed and those where it did not. These findings suggest that *G. pelaensis* has a serious impact on the soil fauna of Ogasawara.

### Feeding habits of *Geonemertes pelaensis*

**Figure 3:**
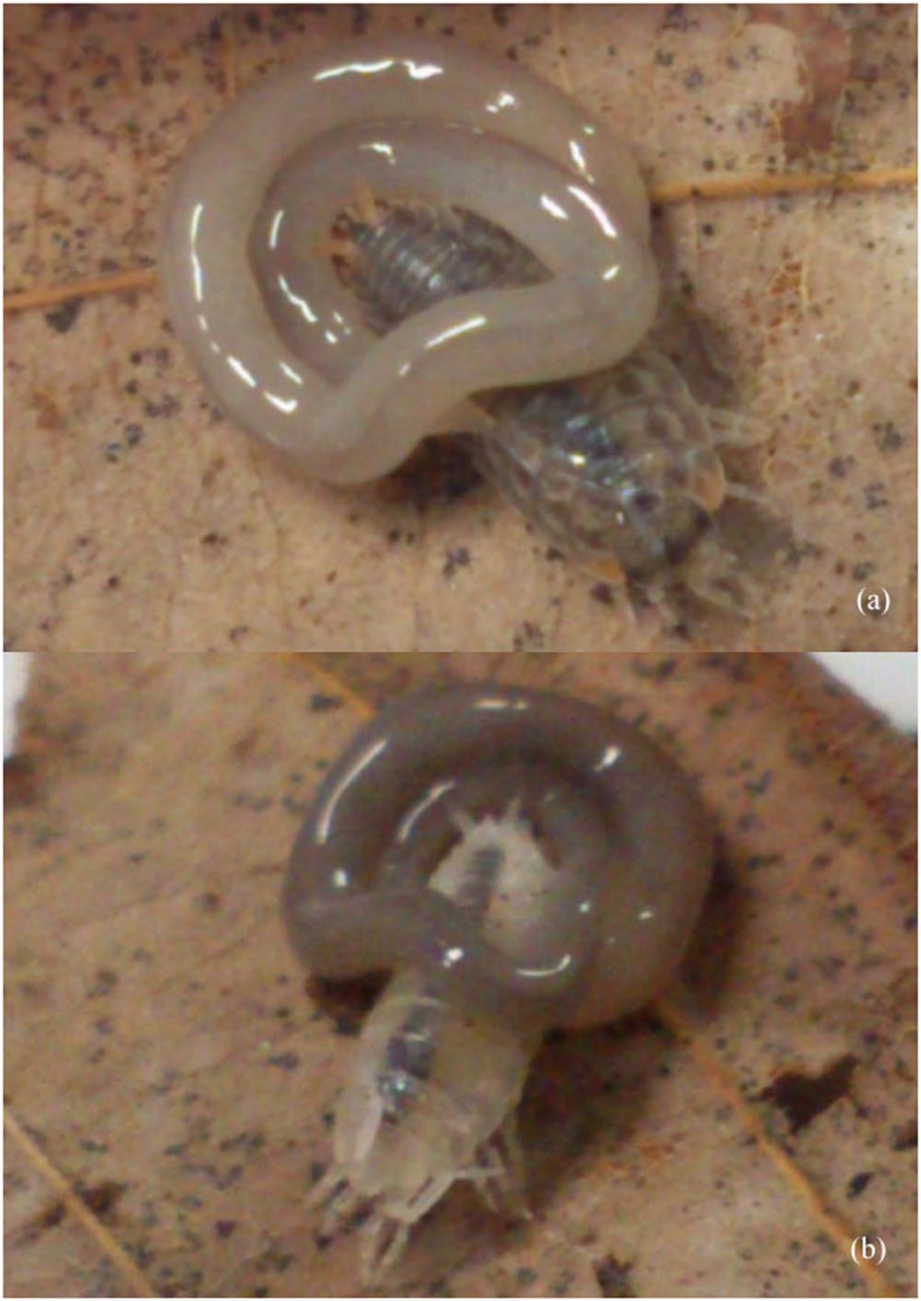
Photographs showing that *G. pelaensis* preys on *Isopoda sp*. Body color of *G. pelaensis* changed from white (**a**) to grey (**b**), because body contents of *Isopoda sp*. moved to *G. pelaensis* by predation.

Based on our observation of feeding behavior, the nemertine *G. pelaensis* extrudes its proboscis when it catches prey and/or is stimulated from the outside—like most other groups of nemertines. In the feeding experiments, *Burmoniscus okinawaensis* (Isopoda), *Spherillo boninensis* (Isopoda) and *Talitridae sp*. (Amphipoda) were predated by *G. pelaensis*, and 2, 4 and 7 individuals were consumed, respectively. In our experiments, we could not identify which individual nemertine consumed each prey item. When *G. pelaensis* preyed on *B. okinawaensis* and *S. boninensis*, it sucked out the internal parts of the victim’s body, resulting in a change of colour from dark grey to white in the victims and from white to dark grey in *G. pelaensis* (Fig. 3). In contrast, *Nerthra macrothorax* (Hemiptera) and *Japanioiulus lobatus* (Diplopoda) were not predated by *G. pelaensis*.

We did not use Araneae and Diptera for the present feeding experiments, because it seemed unlikely that the nemertine could capture animals like these quickly moving animals. However, in our additional experiments, we observed that *G. pelaensis* preyed on a spider (see Supplementary Video S1), *Drosophila*, *Cicadoidea* and *Lepidoptera* (See Supplementary Fig. S1).

## Discussion

The present study documents that the land nemertine *Geonemertes pelaensis* is a predator of arthropods and caused serious declines of Isopoda and Amphipoda in Ogasawara. The decline of arthropod fauna, particularly the disappearance of Isopoda and Amphipoda, that has occurred on Chichijima since the 1980s are results of predation by this non-native nemertine. Similar decreases in land crustaceas formerly recorded in the north and central parts of Hahajima are also caused by predation of this nemertine. These conclusions are supported by our field surveys and feeding experiments. In Ogasawara, Isopoda and Amphipoda are the dominant components of the original soil fauna, and all of the species that belong to Isopoda and Amphipoda are native^21,37^. Approximately 80% of isopod species in Ogasawara are endemic^21^. Amphipoda also includes many endemic species, though the taxonomy of amphipods in Ogasawara remains unclear. These imply that predation by non-native nemertine is a serious conservation issue.

Other orders such as Diplopoda show no decline in the areas where *G. pelaensis* exists, suggesting that *G. pelaensis* does not feed on these groups. The result of our feeding experiment on Diplopoda is consistent with this conclusion. In contrast, *Nerthra macrothorax*, which showed a decline of density in nature but were not predated in the laboratory, reveals an incongruence. It is most likely that the decline of *Nerthra macrothorax* is due to indirect effects of predation by *G. pelaensis*. *Nerthra macrothorax* is carnivorous insect and preys on mainly isopods^38,39^. Therefore, radical decline of isopods caused by the nemertine’s predation subsequently results in declines of *Nerthra macrothorax* because of scarcity of its diet. Declines of carnivorous Chilopoda at the sites where *G. pelaensis* exists also appear to be the result of indirect effects of predation by *G. pelaensis*. Although Chilopoda was not used in the feeding experiments, Chilopoda is unlikely to be predated by *G. pelaensis*, considering the result of the feeding experiment on Diplopoda. Araneae, which is predated by *G. pelaensis*, may have some resistance against predation by *G. pelaensis*, because it is mainly composed of non-native species. In addition, the main diet of Araneae in Minamizaki consists of microorganisms such as Collembola and mites, which are unlikely to be consumed by *G. pelaensis*.

Hence, predation by *G. pelaensis* causes not only serious decreases in the density of Isopods, but it also causes cascade effects on the food webs in the soil ecosystems of Ogasawara. As a result of the nemertine’s predation, biomass of soil fauna radically decreases in Ogasawara, and ecological function of the soil ecosystem is seriously damaged.

The contraction of the distribution of Isopoda from north to south in Minamizaki, which has been reported in the previous studies^27^, appears to reflect the spread of the distribution of *G. pelaensis* from north to south in this area. In our preliminary survey conducted in January, *G. pelaensis* was not recorded in site 4, whereas *G. pelaensis* was recorded at this site in our survey in September, suggesting expansion of the distribution of *G. pelaensis* toward the south.

Distributions of *G. pelaensis* in Ogasawara remain unclear except for the islands of Chichijima, Hahajima and Nishijima. Our preliminary surveys documented that no *G. pelaensis* occurs on the Nishijima Island, which has rich soil arthropod fauna dominated by isopods and amphipods. Further surveys of the distribution of *G. pelaensis* and status of soil fauna are required in other islands of Ogasawara. The development of a quarantine program is required to prevent further spread of *G. pelaensis* to the islands like Nishijima that are not invaded by *G. pelaensis*.

There is a striking contrast in terms of feeding habits of *G. pelaensis* between our findings and a previous study that showed that *G. pelaensis* is malacophagous and does prey on arthropods on the basis of feeding experiments. This inconsistency may imply that *G. pelaensis* includes different cryptic species with different feeding habits. Further taxonomic studies of this land nemertine are required to address this issue. As an alternative hypothesis, shifts in feeding habits have occurred in the populations of Ogasawara, and levels of invasiveness would be different among different ecosystems^12^. Further surveys of the feeding habits of *G. pelaensis* in other regions are needed to clarify this issue. In addition, it is urgently needed to assess the impacts of *G. pelaensis* on native arthropod fauna in other tropical islands where this nemertine has already invaded. Like the case of Ogasawara, unnoticed decline of soil fauna may have already occurred. Furthermore, it is very crucial to prevent further spread of *G. pelaensis* to other regions. This nemertine has expanded its distribution to tropical regions via transport with crops^40^. However, identification of this nemertine is difficult, and this nemertine is easily overlooked. It would be rather efficient and important to develop a system to prevent invasion of any non-native species through transportation of agricultural crops.

The present findings raise an issue and point to a limitation relative to the use of an official black list of invasive species, such as Japan’s IAS list, to anticipate threats of particular non-native species and to prevent their spread. The government generally has no responsibility to monitor IAS risk or to prevent the spread of non-native species that are not on the official invasive species list, particularly if there is no evidence that it potentially exhibits serious risks. In Ogasawara, this caused a substantial delay in recognizing the threats of *G. pelaensis*. The decline of soil arthropods, particularly of isopods and amphipods, has been long believed to be a result of predation by the invasive toad *Bufo marinus*. However, the impact of *G. pelaensis* on the soil arthropod fauna in Ogasawara is far more serious than that of *Bufo marinus* and African big-headed ants *Pheidole megacephala*, both of which are on the list of “100 of the World’s Worst Invasive Alien Species” by IUCN^41^. Any stakeholders using the official list of invasive species to develop conservation programs should recognize that there are always unknown risks related to the invasion of non-native species and that they have information of only a small fraction of non-native species.

## Methods

### Field surveys

The forest at Minamizaki are dominated by *Planchonella obovata*, *Rhaphiolepis wrightiana*, *Calophyllum inophyllum*, *Terminalia catappa*, *Livistona chinensis* and *Pandanus boninensis*. We conducted a soil fauna investigation and survey of the distribution of *Geonemertes pelaensis* during July 4–11 and August 19 to September 17 in 2016. Preliminary surveys of *G. pelaensis* were conducted in January 2016.

Soil arthropod fauna were investigated at 33 sites (Fig. 1c) (9 sites in July, 24 sites in August and September). The study sites were placed so as to cover the entire area of Minamizaki. Each site was 10 m × 10 m in area and placed on the forest floor. Three quadrates (25 cm × 25 cm each) were placed at random within each site. The litter layer and topsoil (up to 5 cm) were collected from each quadrate. Soil animals were collected by hand-sorting them from the collected soil and litter. We excluded mites from subject organisms because they were too small to collect by hand-sorting. The collected animals were preserved by 80% ethanol and were identified under a stereomicroscope. The soil water content was also measured by a meter (model PMS-714, FUSO) in each quadrate.

A survey of the distribution of *G. pelaensis* was conducted at the same 33 sites during the daytime. At each site, we searched for *G. pelaensis* 15 minutes by looking under rotten log, fallen leaves of *Livistona chinensis* and the base of the leaves of *Pandanus boninensis*, and we recorded whether nemertine was distributed. All of these materials of land nemertine were identified as *Geonemertes pelaensis* (Fig. 1), because there was no records of other species of land nemertine in Ogasawara^30^.

### Feeding experiments of *Geonemertes pelaensis*

We conducted feeding experiments of *G. pelaensis* at the Ogasawara Environmental Planning Laboratory, Hahajima. The individuals of *G. pelaensis* were collected from the survey sites of Minamizaki and carried to the laboratory. Five individuals were kept in each plastic box (about 10 cm × 10 cm × 5 cm) for one week with five prey items. The individuals of *G. pelaensis* that were used for the experiment were 2~3 cm length. *Burmoniscus okinawaensis*, *Spherillo boninensis*, *Nerthra macrothorax*, *Talitridae sp* and *Japanioiulus lobatus* were selected for prey items; their lengths were 5 mm, 5 mm, 5 mm, 10 mm and 15~20 mm, respectively. All these prey items were collected from Minamizaki and carried to the laboratory. A wet-paper layer was laid on the bottom of the boxes to prevent desiccation. We counted the number of prey items eaten by the nemertine every day; if organisms were consumed, we added new individuals equal to the consumed number of individuals. This experiment was conducted only for investigating whether *G. pelaensis* can eat arthropods, and so preference of different diets and consumption rate was not surveyed.

### Statistical analysis

The species identified were grouped into orders or classes. To evaluate the impact of *G. pelaensis* on soil fauna, generalized liner mixed models (GLMM) were created using the R library lme4^42^. The number of individuals of each taxa collected from each quadrate was used as the dependent variables (n = 99). The taxa of which the total number of individuals found were less than 50 were not used for GLMM. The taxa used for GLMM included Isopoda, Amphipoda, Diplopoda, Chilopoda, Araneae, Schizomida, Hymenoptera, Coleoptera, Collembola, Diplura, Lepidoptera and *Nerthra macrothorax* (represented Hemiptera). Elevation and water content in each quadrate were used as independent variables. *Pheidole megacephala* is narrowly distributed within the central part of Minamizaki^12^. In order to evaluate its influence separately, presence or absence of this ant was also included as an independent variable. In the analysis of Hymenoptera, presence or absence of *P. megacephala* was not treated as an independent variable because *P. megacephala* was included in the collected samples of Hymenoptera. The presence or absence of *G. pelaensis* and *P. megacephala* was treated as binary data (1: presence, 0: absence). The dependent variable is assumed to have a Negative binomial error distribution. This error distribution has been used to describe over-dispersed datasets or count data of soil animals^43–45^. All GLMMs included individuals across the sites, and the difference of the quadrates was treated as random effects. Stepwise backward selection method was used to determine selected model. Non-significant independent variables (P > 0.05) were removed from full model one at a time. P-values from GLMMs were obtained by model comparisons (Chi-squared test).

## Electronic supplementary material

Supplementary information

Supplementary Video S1
