# Supplementary material for: Declining soil Crustacea in a World Heritage Site caused by land nemertean
Source: Sci Rep. 2017 Sep 29;7:12400. doi: 10.1038/s41598-017-12653-4 (PMC5622052; doi:10.1038/s41598-017-12653-4)
Supplement: Supplementary file 1 — Supplementary information [file 41598_2017_12653_MOESM1_ESM.pdf]

Title: Declining soil Crustacea in a World Heritage Site caused by land nemertean

Author: Shotaro Shinobe, Shota Uchida, Hideaki Mori, Isamu Okochi, Satoshi Chiba

	Intercept		*Geonemertes pelaensis*			*Pheidole megacephala*			Water content			Elevation		
	Estimate	S.E.	Estimate	S.E.	p-value	Estimate	S.E.	p-value	Estimate	S.E.	p-value	Estimate	S.E.	p-value
Isopoda	3.9069	0.3238	-2.875	0.5601	7.68E-06									
Amphipoda	0.5284	0.3986	-4.366	1.014	3.35E-06									
Chilopoda	-0.211	0.1558	-0.664	0.3148	0.03019									
Hemiptera	-1.138	0.3854												
Nerthra macrothorax	-1.036	0.4825	-2.406	1.0472	0.0071									
Collembola	-3.12	0.5196	1.6797	0.3724	5.05E-05				0.1081	0.0271	0.0018			
Scizomida	-0.304	0.2014				-1.288	0.5476	0.01235						
Lepidoptera	-1.374	0.3564				1.7902	0.6003	0.005731						
Hymenoptera	2.4791	0.1227												
Araneae	1.2135	0.1283												
Coleoptera	0.0588	0.1576												
Diplopoda	1.1466	0.3626												
Diplura	-0.527	0.2585												

Supplementary Table S1

The result of generalized liner mixed model (GLMM) demonstrating the relationship between environmental factors and number of individuals in each taxonomic group at each study quadrates (n=99).

Stepwise backward selection method was used. Non-significant independent variables ($P>0.05$) were removed from full model one at a time.

P-values from GLMMs were obtained by model comparisons (Chi-squared test). S.E. indicates standard error.

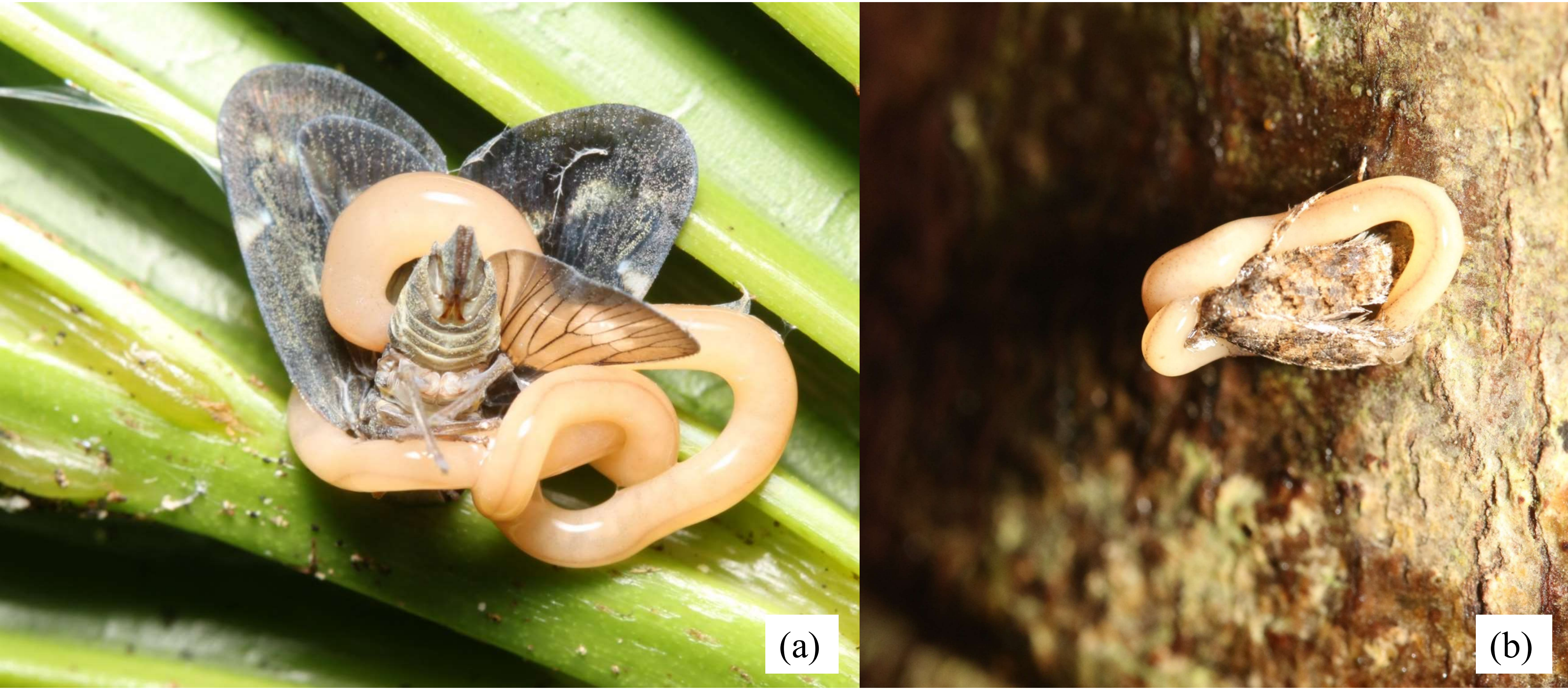

Supplementary Figure S1
Attack of land nemertine to (a) *Cicadoidea sp.* (b) *Lepidoptera sp.*.

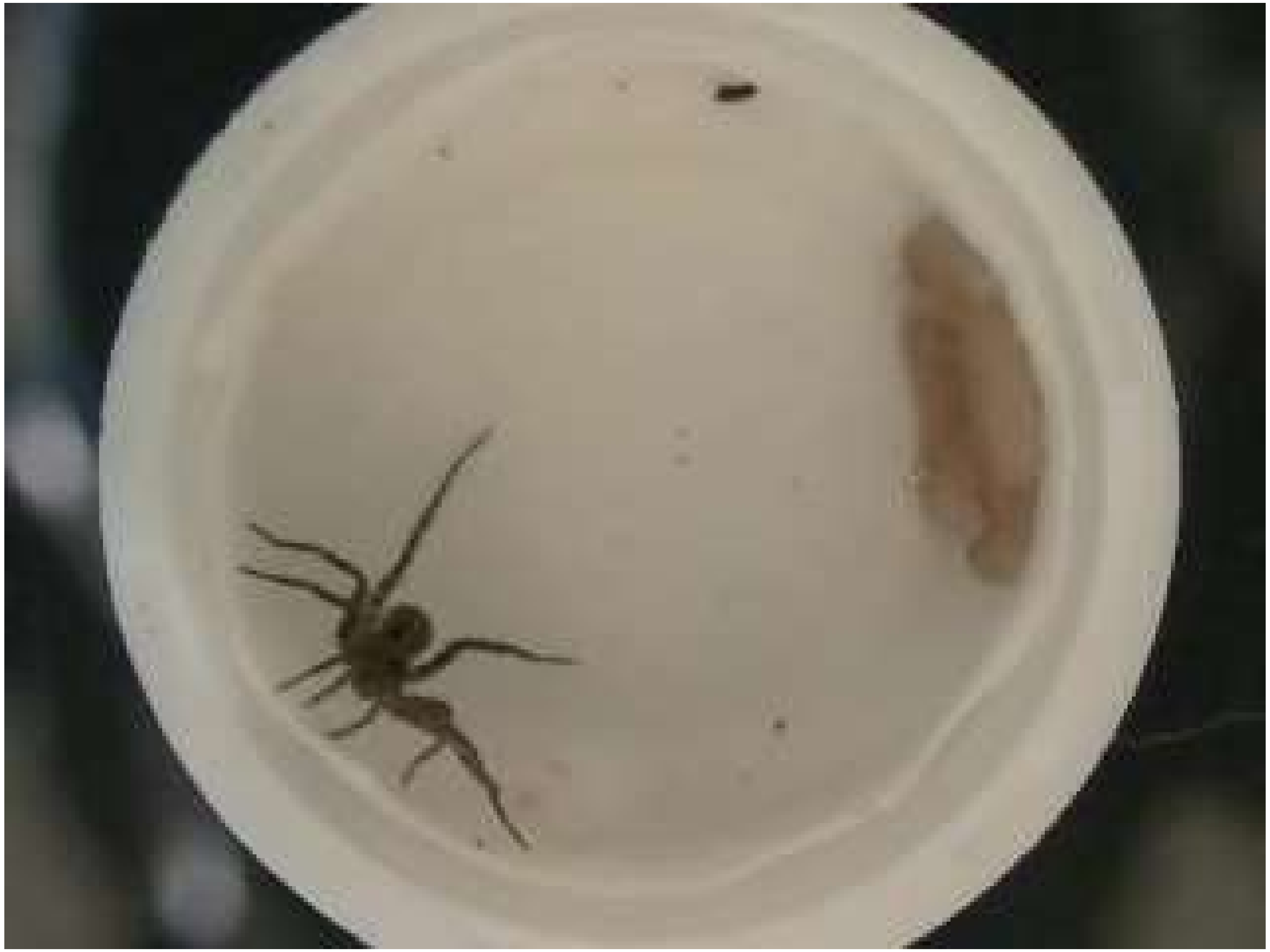

Supplementary Video S1
Attack of land nemertine, *Geonemertes pelaensis* on a spider.
